# Severe disseminated VZV in an immunocompromised host temporally associated with recent recombinant adjuvanted herpes zoster vaccination

**DOI:** 10.1016/j.idcr.2026.e02673

**Published:** 2026-07-07

**Authors:** Raphaël Clement, Ewa Wierzbicka-Hainaut, Julien Mahe, Damien Boutin

**Affiliations:** a Department of Dermatology, CHU de Poitiers Site de la Milétrie, Poitiers, France; b Department of Pharmacovigilance, CHU de Poitiers Site de la Milétrie, Poitiers, France

**Keywords:** VZV reactivation, Shingrix, Immunosuppression

## Abstract

**Background:**

Recombinant adjuvanted herpes zoster vaccine (Shingrix) effectively prevents varicella-zoster virus (VZV) reactivation and is recommended for older and immunocompromised adults.

**Case presentation:**

We report a severe early disseminated VZV reactivation occurring 14 days after primary Shingrix vaccination in an 80-year-old man with a history of lymphocytic lymphoma in biological relapse and secondary hypogammaglobulinemia. Initial presentation featured multimetameric necrotic and hemorrhagic thoracic lesions, which progressed despite oral valaciclovir to a diffuse cephalocaudal vesicular eruption with fever and severe pain, prompting hospitalization. Workup identified VZV-associated bone marrow involvement with acute anemia requiring transfusion; PET imaging excluded lymphoma transformation. The patient improved under intravenous acyclovir (10 mg/kg every 8 h) with daily local care, followed by extended valaciclovir prophylaxis. Given the severity and timing, the second Shingrix dose was withheld.

**Discussion:**

Causality is difficult to establish: primary varicella could not be fully excluded, and vaccine-induced immunity would not yet be established after a single early dose. Nonetheless, the short latency, immunocompromised status, and concordance with rare reports of early post-vaccination zoster suggest a possible association. This case highlights the importance of reporting to pharmacovigilance systems and differentiating adverse events from vaccine failure, without undermining the overall favorable safety profile of Shingrix in high-risk populations.

## Introduction

Vaccination with the recombinant adjuvanted herpes zoster vaccine, Shingrix, received marketing authorization in France in 2018 for the prevention of varicella-zoster virus (VZV) reactivation in adult patients [1]. The vaccination schedule consists of two intramuscular injections administered 2 months apart in order to achieve vaccine-induced immunity. Vaccination with Shingrix has demonstrated an excellent safety profile, including in immunocompromised patients, who represent the new target population compared with the live attenuated vaccine. However, rare cases of varicella-zoster virus (VZV) reactivation have been reported following vaccination with Shingrix, raising the question of a potential association.

We report a case of severe early disseminated VZV reactivation following primary vaccination with Shingrix.

## Case report

**Fig. 1:**
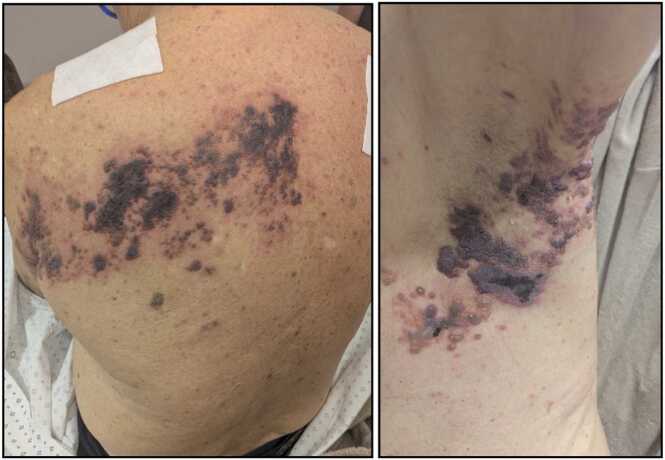
Initial clinical presentation: left-sided thoracic necrotic and hemorrhagic multimetameric lesions.

An 80-year-old patient with a history of lymphocytic lymphoma diagnosed in 2015, who had received first-line polychemotherapy (Rituximab/Fludarabine/Cyclophosphamide) followed by sustained remission since 2016, presented with a biological relapse since 2025 under simple monitoring, associated with secondary hypogammaglobulinemia. Fourteen days after primary vaccination with the recombinant adjuvanted herpes zoster vaccine, Shingrix, the patient developed VZV reactivation with necrotic and hemorrhagic multimetameric thoracic involvement (Fig. 1). The patient’s immunological status regarding varicella-zoster virus was unknown.

**Fig. 2:**
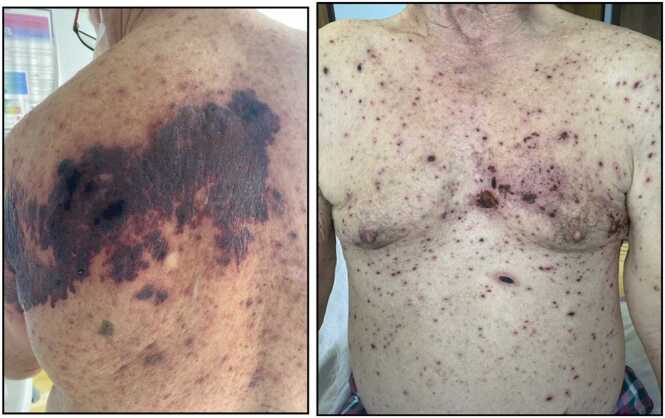
Clinical presentation on admission: worsening of the multimetameric thoracic lesions and appearance of diffuse vesicular/post-vesicular lesions.

The initial course was unfavorable despite treatment with Valaciclovir, with the development of a diffuse vesicular eruption beginning in the cephalic region and subsequently spreading downward, associated with high fever and severe pain, leading to hospitalization in our department (Fig. 2).

VZV-associated bone marrow involvement was identified in the context of acute anemia requiring transfusion support. Transformation into high-grade lymphoma was ruled out following an unremarkable PET scan.

**Fig. 3:**
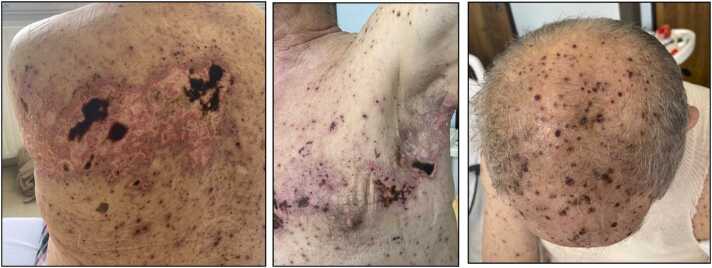
Clinical presentation at hospital discharge: drying and regression of the different lesions.

Subsequently, the clinical course became favorable under intravenous Aciclovir administered at a dosage of 10 mg/kg every 8 h, combined with daily local wound care (Fig. 3). Prolonged prophylactic treatment with Valaciclovir was initiated after multidisciplinary discussion with the patient’s referring hematologist. Furthermore, as a precautionary measure given the severity of the presentation, administration of the second dose of Shingrix was contraindicated. The patient did not develop post-herpetic neuralgia following the infectious episode.

## Discussion

The recombinant herpes zoster vaccine, Shingrix, is an adjuvanted subunit vaccine targeting glycoprotein E of the varicella-zoster virus. It has largely replaced the live attenuated vaccine, Zostavax, which was contraindicated in two major populations at high risk of herpes zoster reactivation: immunocompromised patients and adults older than 75 years [1].

The efficacy of the recombinant adjuvanted herpes zoster vaccine against herpes zoster has been estimated between 68.2% and 87.2% in clinical trials involving immunocompromised adults [2], [3].

The risk of early post-vaccination herpes zoster is not mentioned in the Summary of Product Characteristics for the vaccine.

Several cases of herpes zoster following vaccination with the recombinant adjuvanted herpes zoster vaccine have been reported. In a phase IV study, Hesse et al. reported 196 cases of herpes zoster (4.5%) among 4381 adverse events following vaccination with Shingrix, although no temporal data between viral reactivation and vaccination were provided, thereby limiting assessment of causality [4]. Four individually reported cases described short delays between vaccination and viral reactivation (ranging from 1 to 7 days), most often in patients with comorbidities or immunosuppression (Table 1) [5], [6], [7], [8]. All individually reported cases described localized herpes zoster in patients with either systemic or local immunosuppression. In the French pharmacovigilance database, a case of disseminated herpes zoster occurring 6 days after primary vaccination has also been reported, complicated by pancytopenia and primary herpes simplex infection in an immunocompromised patient receiving long-term methotrexate therapy for rheumatoid arthritis.

**Table 1:** Medical and demographic characteristics of the four cases of early varicella-zoster virus reactivation following vaccination with Shingrix reported in the literature [5], [6], [7], [8].

**Reference**	**Gender**	**Age**	**Comorbidities**	**Localization**	**Time to onset**
Housel (2020)	F	32	Lupus treated with hydroxychloroquine, azathioprine and prednisone	Thoracic	1 day
Mittal (2022)	F	73	Breast cancer	Thoracic	3 days
Lu and al (2022)	F	75	Glaucoma	Keratitits	2 weeks
Altukheim (2023)	F	60	Type 2 diabetes	Thoracic	1 week

The interpretation of this case requires consideration of three possible explanations for VZV reactivation following administration of the recombinant adjuvanted herpes zoster vaccine: a vaccine-associated adverse event, a spontaneous viral reactivation unrelated to vaccination, or incomplete vaccine-induced protection. Distinguishing between these possibilities is particularly challenging in immunocompromised patients, who remain at high baseline risk of herpes zoster despite vaccination.

Several factors preclude establishing a causal relationship between vaccination and VZV reactivation. Based on the clinical presentation, the patient had most likely experienced prior varicella infection; however, in the absence of documented evidence, primary varicella cannot be formally excluded, although this remains unlikely. It should also be emphasized that Shingrix is not indicated for the prevention of primary varicella infection. Furthermore, a coincidental viral reactivation occurring after the first vaccine dose, before protective immunity had developed, cannot be excluded.

However, several observations support a possible vaccine-associated event. The short interval between vaccination and symptom onset is consistent with the few published reports of early herpes zoster following Shingrix vaccination, although all previously reported individual cases involved localized disease. Although, isolated case reports cannot establish causality, the consistency of this temporal pattern across reports supports continued pharmacovigilance surveillance.

Incomplete vaccine protection should also be considered when herpes zoster develops after vaccination. This explanation is, however, more plausible when infection occurs after sufficient time has elapsed for vaccine-induced immunity to develop or after completion of the recommended vaccination schedule. In the present case, disseminated VZV developed shortly after the first vaccine dose, making incomplete vaccine protection a less likely explanation.

The pathophysiological mechanisms underlying early VZV reactivation after Shingrix remain uncertain. Proposed hypotheses include transient immune dysregulation or an exaggerated inflammatory response induced by the vaccine adjuvant, although these mechanisms remain speculative [7].

This case also emphasizes the importance of pharmacovigilance. Early herpes zoster occurring within approximately two weeks after Shingrix vaccination should be systematically reported, particularly in immunocompromised patients, to better characterize the frequency, clinical spectrum, and potential causality of these rare events. Based on the currently available literature, the longest interval between vaccination and a potentially vaccine-associated VZV reactivation is 14 days (range, 2–14 days). In contrast, herpes zoster occurring beyond this interval is more likely to reflect spontaneous reactivation or incomplete vaccine protection than a vaccine-triggered adverse event.

We report the first case of severe early disseminated VZV reactivation temporally associated with the recombinant adjuvanted herpes zoster vaccine in an immunocompromised patient. Although causality cannot be established, this case illustrates the diagnostic challenge of distinguishing a vaccine-associated adverse event from coincidental infection or incomplete vaccine protection. Systematic pharmacovigilance reporting of early post-vaccination VZV reactivation will be essential to better define the characteristics and clinical significance of these uncommon events while preserving the well-established favorable benefit-risk profile of the recombinant herpes zoster vaccine.

## CRediT authorship contribution statement

**Julien Mahe:** Resources, Investigation. **Ewa Wierzbicka-Hainaut:** Validation, Supervision. **Damien Boutin:** Visualization, Validation, Supervision. **Raphaël Clement:** Writing – review & editing, Writing – original draft, Validation, Methodology, Conceptualization.

## Consent

Written informed consent was obtained from the patient for publication of this case report and accompanying images.

## Funding

There was no specific funding for this study.

## Declaration of Competing Interest

The authors declare that they have no known competing financial interests or personal relationships that could have appeared to influence the work reported in this paper.
